# Perceptions, use and attitudes of pharmacy customers on complementary medicines and pharmacy practice

**DOI:** 10.1186/1472-6882-10-38

**Published:** 2010-07-20

**Authors:** Lesley A Braun, Evelin Tiralongo, Jenny M Wilkinson, Ondine Spitzer, Michael Bailey, Susan Poole, Michael Dooley

**Affiliations:** 1 Cardiothoracic Surgical Research Unit, Department of Surgery, Monash University, Alfred Hospital, Melbourne, Australia; 2 Department of Pharmacy, Alfred Hospital, Melbourne, Australia; 3 Department of Pharmacy, Griffith University, Gold Coast, QLD, Australia; 4 School of Biomedical Sciences, Charles Sturt University, Wagga Wagga, Australia; 5 Faculty of Pharmacy and Pharmaceutical Sciences, Monash University, Parkville, Australia; 6 Department of Epidemiology and Preventive Medicine School Public Health & Preventive Medicine, Monash University Alfred Hospital, Melbourne, Australia

## Abstract

**Background:**

Complementary medicines (CMs) are popular amongst Australians and community pharmacy is a major supplier of these products. This study explores pharmacy customer use, attitudes and perceptions of complementary medicines, and their expectations of pharmacists as they relate to these products.

**Methods:**

Pharmacy customers randomly selected from sixty large and small, metropolitan and rural pharmacies in three Australian states completed an anonymous, self administered questionnaire that had been pre-tested and validated.

**Results:**

1,121 customers participated (response rate 62%). 72% had used CMs within the previous 12 months, 61% used prescription medicines daily and 43% had used both concomitantly. Multivitamins, fish oils, vitamin C, glucosamine and probiotics were the five most popular CMs. 72% of people using CMs rated their products as 'very effective' or 'effective enough'. CMs were as frequently used by customers aged 60 years or older as younger customers (69% vs. 72%) although the pattern of use shifted with older age.

Most customers (92%) thought pharmacists should provide safety information about CMs, 90% thought they should routinely check for interactions, 87% thought they should recommend effective CMs, 78% thought CMs should be recorded in customer's medication profile and 58% thought pharmacies stocking CMs should also employ a complementary medicine practitioner. Of those using CMs, 93% thought it important for pharmacists to be knowledgeable about CMs and 48% felt their pharmacist provides useful information about CMs.

**Conclusions:**

CMs are widely used by pharmacy customers of all ages who want pharmacists to be more involved in providing advice about these products.

## Background

Herbal medicines, nutritional and dietary supplements, also known as complementary medicines (CMs), have become increasingly popular in the United States, United Kingdom, Canada and Australia, with self-medication making up the majority of use [1-5].

While it is difficult to provide exact data on the use of complementary medicine in Australia it is clear that a significant proportion (up to 75%) of the Australian public have used complementary medicine in a number of different forms [2,6,7]. Data from the Australian Bureau of Statistics shows an 80% increase in people employed as CM practitioners in the 10 years to 2006; in the same time period the number of people visiting CM practitioners within a 2 week period rose from approximately 500,000 to 750,000 [8]. Together with data from a national survey which estimated that 69% of Australian adults used at least one CM product and/or visited a CM practitioner in the previous 12 months, there is little doubt about the impact of CMs in Australian health care [2].

Community pharmacy is one of the main suppliers of CM products in Australia and is the primary outlet for approximately 40% of the total money spent on this sector which has been estimated at between AU$800 million and at AU$1.86 billion dollars annually [2,7]. Several studies have investigated the attitudes and opinions of pharmacists and pharmacy students towards the use of CMs [9-13] however the attitudes, use and expectations of Australian pharmacy customers have not yet been examined. This is important to investigate as the widespread use of CMs is likely to have raised pharmacy customer needs and expectations of pharmacists' responsibilities with respect to CMs.

Within the community pharmacy setting, pharmacists and pharmacy assistants are in a position to provide guidance about consumer purchases of over the counter medicines. Indeed, as primary care providers, pharmacists have a professional obligation to provide information and guidance to patients about the quality use of all medicines, which, according to the Australian National Medicines Policy, includes CMs [14].

The primary aims of this project were to investigate the perceptions, use and attitudes of pharmacy customers to CMs and explore their expectations of pharmacy practice. Secondary aims were to compare patterns of CM use between younger and older (over 60 years) pharmacy customers.

## Methods

An anonymous, self-administered questionnaire was developed to collect data from pharmacy customers (Additional file 1; Pharmacy customer CAM survey). The questionnaire adopted and adapted questions from other surveys [2,7,15,16] and included new questions relevant to the study. An advisory committee consisting of three pharmacists and a consumer advocate also informed survey development. The questionnaire was pre-tested using a convenience sample of 40 randomly selected pharmacy customers and then revised before wider utilisation.

The survey consisted of 32 core questions which collected demographic, behavioural, health and attitudinal information. An additional 16 questions were asked of customers who reported using CMs. These questions focussed specifically on issues relating to CMs including which products were used, purchase locations, product effectiveness and attitudes to pharmacy practice as it currently relates to CMs. Response options varied depending on the type of question asked and included multiple choice, open-ended free text and Likert-scaled responses.

Three project sites were selected to provide information from 60 pharmacies located in metropolitan and regional areas of Australia. Melbourne (Victoria) and the Gold Coast (Queensland) were the metropolitan sites; Wagga Wagga (New South Wales) was the regional site. These locations were selected due to convenience for recruitment due to proximity to the investigators academic location. A representative population sample of 1000 customers was targeted based on approximately 50% expected to be using CMs.

Pharmacy sites used for customer data collection were randomly chosen using the pharmacy listing in a national telephone directory. Pharmacy managers were contacted and those that agreed for their site to be involved in the data collection phase were visited by a dedicated research assistant who randomly selected pharmacy customers for recruitment as they exited the stores. Customers were then approached by a research assistant in the pharmacy and asked if they would like to participate in a survey. Customers filled out the surveys on site before leaving the pharmacy.

All data were entered into an electronic version of the survey which was available through SurveyMonkey™, an on-line survey tool. Ethics approval was obtained from the Alfred and Monash Human Research Ethics Committee, and subsequently from Charles Sturt and Griffith University.

### Data analysis

Descriptive and inferential statistics were calculated using SAS version 9.1 (SAS Institute Inc., Cary, NC, USA). Differences in proportions between groups were compared using chi-square tests for independent proportions or Fishers Exact tests where numbers were small. Continuous, normally distributed variables were compared using Student's t-test and reported as means (standard deviations), while non-normally distributed variables were compared using Wilcoxon Rank Sum tests and reported as medians (interquartile range). To reduce the chance of type I errors, a reduced p-value of 0.05 was considered to be statistically significant.

## Results

A total of 1,121 pharmacy customers completed questionnaires (response rate 62%), of which 65% (n = 728) were from metropolitan Melbourne, 27% (n = 307) from the Gold Coast region and 8% (n = 86) from Wagga Wagga. Data were not collected on patient who declined to be involved. Of the 54 pharmacies that were involved, there were 30 from Melbourne, 16 from the Gold Coast and 8 from Wagga Wagga. More women participated in the survey than men (74% vs. 25%) and participants ranged in age from 18 years to over 70 years with a broad range in between. Further demographic and baseline data are presented in Table 1.

**Table 1:** Demographic data of pharmacy customers

		N (%)*
Gender	Male	275 (25)
	Female	805 (74)
	Not reported	41 (4)
	Total number of respondents	1121 (100)
Highest level of education attained	Did not go to school	6 (0.5)
	Secondary education	343 (31)
	Certificate level	218 (20)
	Diploma and advanced diploma level	137 (13)
	Bachelor degree	187 (17)
	Graduate diploma or certificate	67 (6)
	Postgraduate degree	110 (10)
	Not reported	24 (2)
Current work status	Employed full time	327 (30)
	Self employed	108 (10)
	Not in the labour force	330 (30)
	Employed part time	241 (22)
	Unemployed	60 (6)
	Not reported	26 (2)
Current age (years)	15-19	23 (2)
	20-29	162 (15)
	30-39	184 (17)
	40-49	187 (17)
	50-59	208 (19)
	60-69	177 (16)
	Over 70	142 (13)
	Not reported	9 (1)

• % of total respondents

### Self reported health of customers

Nearly half (47%) of participants reported their health as good, 36% as very good and 11% as excellent, with 5% reporting it as poor. Hypertension was reported by 24% of customers, arthritis by 20%, high cholesterol levels by 16%, asthma by 11.5% and diabetes by 5%.

Age comparisons reveal a significantly greater proportion of customers aged 60 years or older reported having hypertension (50% vs 13%; p < 0.0001), arthritis (48% vs 9%; p < 0.0001), hyperlipidaemia (30% vs 10%; p < 0.0001) or diabetes (11% vs 3%; p < 0.0001) compared with younger customers. No difference in frequency of asthma was found.

### Medication use

Of the total sample, 61% reported using prescription medicines on a daily basis with 37% using one medicine, 36% using two to three medicines, 16% using four to five medicines and 9% using more than five medicines daily. A significantly higher proportion of men reported using prescription medicines daily than women (25% vs. 16%; p = 0.0009) and significantly higher proportion of people aged 60 years or older took prescription medication daily compared to younger people (87% vs. 48%; p < 0.0001).

CMs had been used by 72% of pharmacy customers (n = 787) in the previous 12 months with significantly more women having used CMs than men (76% vs. 58%; p < 0.0001). There was no significant difference in usage between younger customers and those aged 60 years or older (72% vs 69%). Of the people using CMs, 43% were also taking prescription medicines on a daily basis and 72% rated their CMs as 'very effective' or 'effective enough'.

The ten most popular CMs taken by pharmacy customers are listed in Table 2.

**Table 2:** CMs used by pharmacy customers in the previous 12 months

List of Top 10 CMs taken	N (%)*
Multivitamins	392 (49)
Fish oil supplements	379 (47)
Vitamin C	244 (31)
Glucosamine	234 (29)
Vitamin B complex	197 (25)
Probiotics	134 (17)
Echinacea	94 (12)
Coenzyme Q10	57 (7)
Ginkgo biloba	41 (5)
St Johns wort	37 (5)
**Total customers responding**	**801**

*% of total respondents taking CMs

Age comparisons revealed a greater proportion of people over 60 years had been taking glucosamine (33% vs. 16%; p < 0.0001) or fish oil supplements (41% vs. 32%; p = 0.005) in the previous 12 months compared to younger people whereas significantly fewer in the older age category were using multivitamins (41% vs 22%; p < 0.0001), vitamin C supplements (25% vs 15%;p = 0.0002), probiotics (15% vs 6%; p < 0.0001) or echinacea (11% vs 4%; p= 0.0002).

Respondents were asked who had recommended CMs to them, with most people reporting they had self prescribed (Table 3).

**Table 3:** Recommendation of CMs to pharmacy customers

Recommendation by:	Number (%)*
Myself	335 (42)
Medical doctor	255 (32)
Family/friends	162 (20)
Naturopath/herbalist	160 (20)
Pharmacy assistant	100 (13)
Health food store staff	50 (7)
Pharmacist	81 (10)
Other	48 (6)
**Total responses**	**801**

*% of total respondents answering question

**Figure 1:**
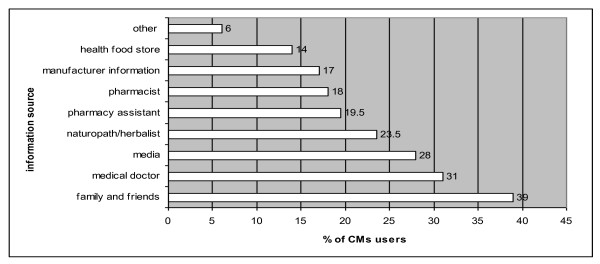
**Sources of CM information used by pharmacy customers (N = 787)**.

Pharmacists were not the main sources of information for pharmacy customers to learn about CMs. Friends and family, medical doctors, the media and naturopaths/herbalists were more often referred to for information (Figure 1).

### Consulting practitioners

Most (93%) participants had seen a medical practitioner within the previous 12 months. Far fewer (39%) participants had seen a CM practitioner in the last 12 months. Of those who had, 38% saw a massage therapist, 33% a naturopath, 31% a chiropractor, 16% an acupuncturist, 12% an osteopath, 9% a Chinese medicine practitioner, 4% a herbalist and 3% a homeopath. Gender and age differences were identified with significantly more women having consulted a CM practitioner than men (43% vs. 27%; p < 0.0001) and a significantly greater proportion of younger customers consulting CM practitioners than those aged 60 years and older (45% vs 25%; p < 0.0001).

### Customer expectations of pharmacy practice

Attitudinal questions were asked of the total sample (CMs users and non-users) about pharmacy practice. The overwhelming majority (92%) indicated that pharmacists should provide safety information about CMs and 90% thought they should routinely check whether CMs interact with prescription medicines. Additionally, 87% thought pharmacists should recommend CMs if they are effective and 78% thought pharmacists should record CMs taken by customers in their medication profile. Furthermore, 58% thought pharmacies which stock CMs should also employ a CM practitioner. In regards to CMs specifically, 87% of customers thought effective CMs should have a 'tick of approval' from a recognised government body with CM expertise and 82% wanted more detailed product information similar to prescription medicine, for all CMs.

Pharmacy customers using CMs were asked more specific attitudinal questions relating to pharmacy practice. (Table 4).

**Table 4:** Pharmacy customers using CMs: attitudes to the pharmacists role in provision of CMs

	Responses				
	Strongly agree	Agree	Neither agree or disagree	Disagree	Strong disagree
	N (%)*	N (%)	N (%)	N (%)	N (%)
My pharmacist is fully aware of any CMs I use	79 (11)	164 (22)	**193 (26)**	192 (25)	120 (16)
I feel comfortable telling my pharmacist about my use of CMs	165 (22)	**372 (50)**	140 (19)	46 (6)	19 (3)
It is important for pharmacists to be aware of the CMs people use	290 (39)	**337 (46)**	91 (12)	21 (3)	3 (0)
My pharmacist encourages questions about CMs	88 (12)	201 (27)	**295 (40)**	112 (15)	37 (5)
My pharmacist provides useful information about CMs	103 (14)	250 (34)	**261 (36)**	91 (12)	27 (4)
I think it is important for a natural medicine practitioner to be located in a pharmacy where they sell CMs	179 (24)	**300 (41)**	185 (25)	63 (9)	11 (1)
I trust my pharmacists' advice about CMs	156 (21)	**348 (47)**	160 (22)	57 (8)	17 (2)
It is important for pharmacists to be knowledgeable about CMs	299 (41)	**383 (52)**	44 (6)	10 (1)	2 (0)
Pharmacy assistants give me more advice about CMs than my pharmacist	61 (8)	204 (28)	**332 (45)**	111 (15)	26 (4)
My pharmacist does not give me information about CMs	40 (5)	145 (20)'	**281 (39)**	197 (27)	66 (9)

## Discussion

The primary aims of this project were to investigate the use, perceptions and attitudes of pharmacy customers to CMs and explore their expectations of pharmacists. CMs are popular amongst pharmacy customers with nearly 3 out of 4 reporting use of a CM product within the previous 12 months. Nutritional supplements feature most prominently, in particular multivitamins, fish oils, vitamin C, vitamin B complex and probiotics. Herbal medicines were less commonly used than nutritional supplements and of these, echinacea, ginkgo, St Johns wort and valerian were most popular. This pattern of use (popularity of vitamin C, multivitamins and fish oil supplements) is consistent with that reported in other Australian studies [16,17].

This study further establishes that CMs remain popular in the older age group although the pattern of use shifts with advancing age. The higher use of glucosamine or fish oil supplements in people aged 60 years or older is not surprising given the increased prevalence of hypertension, osteoarthritis and hyperlipidemia in this group and the established role of fish oils in cardiovascular disease [18] and potential benefits of glucosamine in osteoarthritis [19].

### Pharmacy customers want more from pharmacists

Market research, and more recently health services and social science research, has adopted the concept of the 'new consumer' to describe customers or patients who are becoming more demanding [20,21]. They tend to be information strong (well-informed) and information seeking (inquisitive); ask critical questions; show a desire to initiate dialogue; seek counselling and in general no longer blindly accept the authority of health care providers.

Given the consumer-driven development towards holistic and integrative healthcare [22] some of the findings of this study are not surprising; for example, consumers acceptance of CM integration into pharmacy and their expectation for more interaction with pharmacists in this regard. Nearly all customers expect pharmacists to be knowledgeable about CMs and recommend CMs which are effective, to provide safety information, screen for drug-CM interactions and record patients' use of CMs in their medication profile. A small Canadian study also found customers want pharmacists to be knowledgeable about CMs and take on an advisory role to help them identify and assess information about CMs, and in particular, for them to provide guidance about safety issues [23].

Whilst customers want more engagement with pharmacists regarding CM issues, some customers currently feel that pharmacists are ill-equipped to counsel them about CMs and many do not refer to pharmacists as an information source. This correlates with pharmacists' own discomfort dealing with CM queries and feeling insufficiently informed about CMs [24-26]. It is possible that customer's interest in having access to a natural medicine practitioner within the pharmacy premises is a consequence of their current dissatisfaction with pharmacy practice, however this remains to be further investigated.

### Reducing clinical risk with CMs

CMs, like all medicines, have the potential to cause adverse reactions. Reference texts and herbal pharmacopoeias give detailed information about adverse effects associated with CMs and pharmacovigilance systems collect thousands of adverse reaction reports each year [27-29]. Unlike prescription medicines, CMs are often taken on the advice of family, friends or self determination and, as was observed in this study, are sometimes used in combination with pharmaceutical drugs.

Patient counselling can improve patient safety however in order for this to take place, customers that are interested in or currently using CMs must be willing to engage in discussion about these treatments with healthcare providers who in turn, must have sufficient interest and knowledge to provide an informed opinion about their safe and appropriate use.

It is often reported that people using CMs do not routinely disclose use to their medical practitioner. According to a review of 12 studies, the rate of non-disclosure of those using CM is as high as 77% in some studies [30]. The main reasons patients provide for not disclosing use to their medical practitioners are concerns about a negative response by the practitioners, the belief that the practitioner does not need to know about their CM use, and the fact that the practitioner does not ask. Pharmacy customers in this study did not display the same reticence about discussing CMs with pharmacists and the majority think it is important for pharmacists to be aware of the CMs being taken. This presents pharmacists with an opportunity to step up and take on a greater advisory role which would be welcomed by customers and improve patient safety.

Like all studies, this one has limitations which influence the generalisability of the findings. Self-administered surveys conducted in English are more likely to appeal to people with an interest in the topic being investigated and will limit participation to those people with stronger opinions about the issues being investigated and those that read and write English. Limitations also include the validity and reliability of self reported data. Whilst efforts were taken to recruit participants from different geographical locations at different times of the week in different types of pharmacies, it is possible that the customers participating in the study do not fully represent all pharmacy customers. Nevertheless the comparability of some of the data to other published reports of CM use by the Australian general public suggests that this is not a significant factor.

## Conclusion

Most pharmacy customers have used or are using CMs and expect pharmacist to provide advice about CMs as part of pharmacy practice. Older customers (over 60 years) are also using CMs and for some products, their use is greater than for younger customers. Overall, people using CMs are satisfied with the results obtained from these products and see them as effective therapeutic agents. The challenge now remains for pharmacy practice to meet the needs of the community by up-skilling pharmacists to enable them to provide the guidance about CMs that customers seek.

## Competing interests

Authors of this manuscript have the following to disclose concerning possible financial or personal relationships with commercial entities that may have a direct or indirect interest in the subject matter of this presentation:

Nothing to disclose for all authors.

## Authors' contributions

LB conceived of the study, was project manager, co-ordinated data collection and chief author of this paper. ET and JW contributed to study design, co-ordinated data collection at their sites, MB was chiefly responsible for statistical analysis, OS, SP and MD aided in study design and all aided in results interpretation and have read and approved the final manuscript.

## Pre-publication history

The pre-publication history for this paper can be accessed here:

http://www.biomedcentral.com/1472-6882/10/38/prepub

## Supplementary Material

Additional file 1 **Appendix**. Customer CAM Survey Click here for file
