# Supplementary material for: Perceptions, use and attitudes of pharmacy customers on complementary medicines and pharmacy practice
Source: BMC Complement Altern Med. 2010 Jul 20;10:38. doi: 10.1186/1472-6882-10-38 (PMC2919443; doi:10.1186/1472-6882-10-38)
Supplement: Additional file 1 — Appendix. Customer CAM Survey [file 1472-6882-10-38-S1.PDF]

customer CAM survey

1. location & type of pharmacy info

* 1. choose pharmacy type where survey distributed

- independant
- banner
- warehouse
- clinic
- shopping centre
- shopping strip
- not reported

2. what is the pharmacy code ?

2. Default Section

* 1. How would you describe your overall health?

- Excellent
- very good
- good
- poor
- prefer not to answer

* 2. Please indicate if you have any of the following medical conditions

- high blood pressure
- asthma
- arthritis
- high cholesterol levels
- diabetes
- none
- not reported

Other (please specify)

* 3. Do you take any prescription medicines on a daily basis?

- No
- Yes
- not reported

3. taking script meds

* 1. How many different script medicines do you take each day?

- one
- two or three
- four or five
- more than five
- NONE
- not reported

# customer CAM survey

* 2. Are you taking any of the following medicines ?

- warfarin
- oral contraceptive
- digoxin
- No
- not reported

4. blank page

5. continue backgnd info

* 1. Have you seen a medical doctor in the past 12 months?

- Yes
- No
- not reported

* 2. Have you seen a complementary or alternative medicine practiitoner in the last 12 months ?

- No
- Yes
- not reported

6. which CM practitioners seen?

* 1. Which CAM practitioner/s have you seen in the kast 12 months?

- naturopath/natural medicine practitioner
- herbaList
- osteopath
- homeopath
- chiropractor
- acupuncturist
- massage therapist
- chinese medicine doctor
- not reported
- other

Other (please specify)

7. type of pharmacy generally visited

* 1. Is the pharmacy where you received this survey your usual pharmacy?

- Yes
- No
- not reported

# customer CAM survey

* 2. PLease indicate which type of pharmacy you usually go to for your medicines

- warehouse-style
- banner group pharnacy
- independant pharmacy
- clinic pharmacy (inside or adjacent to a medical clinic)
- other type
- this is my regular pharnmacy
- not reported

* 3. Have you taken any CAM products in the last 12 months ?

- No
- Yes
- not reported

8. CM users go here

* 1. please indicate which CAM product/s you have used

- multivitamin
- vitamin B
- vitamin C
- ginkgo biloba
- a natural weight loss product
- kava kava
- saw palmetto (serenoa)
- probiotics
- glucosamine
- echinacea
- fish oils
- coenzyme Q10
- black cohosh
- valerian
- St Johns wort(hypericum)
- unsure
- not reported
- other

Other (please specify)

* 2. In general, how much money do you spend on CAM products each month?

- up to $20
- $21-50
- $51-100
- over $100
- not reported

customer CAM survey

* 3. Why do you take CAM products?

- It keeps me healthy and gives me a sense of wellbeing
- to prevent disease
- to treat a specific disease or symptom
- it fits into my way of life
- my health problem is not serious enough to take prescription medicines
- they work better or just as well as other medicines
- they were recommended to me
- it gives me a sense of control over my health
- because they have few side effects
- other reason not listed
- not reported
- other

Other (please specify)

* 4. Who usually recommends the CAM products you take?

- pharmacy assistant
- pharmacist
- .
- naturopath/herbalist
- medical doctor
- friends/family
- health food store staff
- other
- myself
- not reported

Other (please specify)

* 5. How effective are the CAM products you take?

- very effective
- effective enough
- somewhat effective
- not effective/useless
- dont know/unsure
- not reported

* 6. Have you ever experienced an adverse reaction (side effect) to a CAM product?

- No
- Yes
- not reported

9. adverse reaction to CM

* 1. How serious was the reaction?

- severe - required hospitalisation
- moderate - symptoms required a visit to a doctor or other healthcare professional
- mild - no specific treatment was required
- Not reported
- other

Other (please specify)

* 2. Who did you tell about the adverse reaction?

- I didnt tell anyone
- a pharmacist
- health food store
- product manufacturer
- a CAM practitioner
- a doctor
- a pharmacy assistant
- family/friends
- rang a professional help line
- not reported
- other

Other (please specify)

* 3. What did you do about the reaction?

- I stopped using the product which I think caused it
- I reduced the dose I was taking
- I changed to another CAM product
- I sought advice from a healthcare professional
- not reported
- other

Other (please specify)

10. CM information sources

* 1. Where do you find out information about CAM products ? (up to 3 responses)

- family/friends
- other
- pharmacy assistant
- naturopath/herbalist
- media eg TV, magazine, radio
- manufacturer information eg labels, pamphlets
- pharmacist
- medical doctor
- someone in health food store
- not reported

Other (please specify)

* 2. Have you ever noticed the term AUST L on a CAM product label?

- No
- Yes
- not reported

11. AUST L meanings

* 1. What do you think it means ?

- This is an Australian made product
- The product is tested by a government agency for safety
- The product is tested by a government agency for quality
- The product is tested by a governemnt agency for effectiveness
- Another meaning
- I dont know
- not reported

12. purchasing habits

* 1. Where do you generally purchase CAM products?

- Pharmacy
- Health food store
- supermarket
- clinic
- internet
- other
- not reported

Other (please specify)

* 2. What are the main reasons you DONT generally purchase CAM products from a pharmacy?

- It is too expensive
- they dont have the specific product I want
- I get better advice about these products elsewhere
- I dont need pharmacist advice about these products
- It is not conveniant for me
- NOT APPLICABLE

Other (please specify)

# customer CAM survey

* 3. What are the main reasons you generally purchase CAM products from a pharmacy?

- It is cheaper than elsewhere
- They have the specific product i want
- I get better advice about these products from te pharmacy than elsewhere
- Professional advice from a natural therapist is available in the pharmacy
- it is conveniant for me
- NOT APPLICABLE

Other (please specify)

13. opinions about pcy and CAM

# customer CAM survey

* 1. Please tick one box for each statement which best reflects your opinion about that statement

	Strongly agree	agree	neutral	disagree	strongly disagree
My pharmacist is fully aware of any CAMs I use					
I feel comfortable telling my pharmacist about my use of CAMs					
It is important for pharmacists to be aware of the CAMs people use					
My pharmacist encourages questionsd about CAM					
My pharmacist provides useful information about CAMs					
I think its important for a natural medicine practitioner to be located in a pharmacy where they sell CAMs					
I trust my pharmacists advice about CAMs					
It is important for pharmacists to be knowledgable about CAMS					
Pharmacy assistants give me more advice about CAMs than my pharmacist					
My pharmaciost does NOT give me information about CAMs					
I have confidence in CAMs					
I have confidence in non-prescription medicines (eg cold and flu tabs)					
I have confidence in prescription medicines prescribed by my doctor					
Not reported					

# customer CAM survey

* 2. Should pharmacy services improve the way they manage CAMs ?

- Yes
- No
- not reported

Yes (please specify)

* 3. Should pharmacists recommend CAM products if they are effective?

- Yes
- No
- not reported

* 4. Should effective CAM products have have a 'tick of approval' from a recognised government body with CAM expertise ?

- Yes
- No
- not reported

* 5. Who would you prefer to recommend CAM products in the pharmacy?

- pharmacist
- an independent CM practiitoner employed in the store
- all of the above
- pharmacy assistant
- no one
- not reported

* 6. Should pharmacies stop selling CAM products?

- Yes
- No
- not reported

Other (please specify)

* 7. Does you regular pharmacy provide enough information about CAM products?

- Yes
- No
- not reported

* 8. Should more detailed product information, similar to script medicine, be provided for all CAM products?

- Yes
- No
- not reported

* 9. Should pharmacists provide safety information about CAM products?

- Yes
- No
- not reported

* 10. Should pharmacists record which CAM products are being taken by customers in the medication profile?

- Yes
- No
- not reported

# customer CAM survey

* 11. Should pharnmacists routinely check whether CAM products taken by customers interact with any prescription medicines?

- Yes
- No
- not reported

* 12. Should pharmacies which stock CAM products also employ a CM practitioner?

- Yes
- No
- not reported

14. demographic details

* 1. what is your current age?

- 15-19
- 20-29
- 30-39
- 40-49
- 50-59
- 60-69
- over 70 years
- not reported

* 2. What is you gender

- Male
- female
- not reported

* 3. What is your residential postcode?

* 4. What is your marital status?

- married/de facto
- separated/divorced
- never married
- widowed
- not reported

* 5. What is your highest level of education?

- did not go to school
- bachelor degree
- secondary education
- graduate diploma or graduate certificate
- certificate level
- post graduate degree level
- advanced diploma and diploma level
- not reported

* 6. What is your current work status?

- employed full time
- employed part time
- self employed
- unemployed
- not in the labour force
- not reported

* 7. Do you have private health insurance?

- Yes
- No
- not reported

# customer CAM survey

* 8. What is your approximate gross (pre-tax) annual household income?

- less than $20K
- $20 001-$40K
- $40 001- $60K
- $60 001-$100K
- over $100K
- not reported

9. add here any additional comments
